# Changes in sensory characteristics, chemical composition and microbial succession during fermentation of ancient plants Pu-erh tea

**DOI:** 10.1016/j.fochx.2023.101003

**Published:** 2023-11-23

**Authors:** Teng Wang, Ruo-yu Li, Kun-yi Liu, Qiu-yue Chen, Nian-guo Bo, Qi Wang, Yan-qin Xiao, Gen Sha, Si-qin Chen, Xin Lei, Yi Lu, Yan Ma, Ming Zhao

**Affiliations:** a College of Tea Science & College of Food Science and Technology, Yunnan Agricultural University, Kunming, Yunnan 650201, China; b State Key Laboratory of Conservation and Utilization of Bio-resources in Yunnan, Yunnan Agricultural University, Kunming, Yunnan 650201, China; c The Key Laboratory of Medicinal Plant Biology of Yunnan Province, National & Local Joint Engineering Research Center on Germplasm Innovation & Utilization of Chinese Medicinal Materials in Southwestern China, Yunnan Agricultural University, Kunming, Yunnan 650201, China; d College of Wuliangye Technology and Food Engineering & College of Modern Agriculture, Yibin Vocational and Technical College, Yibin 644003, China; e Menghai Dazhuo Tea Co., Ltd., Xishuangbanna, Yunnan 666100, China

**Keywords:** Ancient plants Pu-erh tea, Pile-fermentation, Sensory characteristics, Chemical composition, Microbial succession

## Abstract

“Ancient tea plants” are defined as tea trees > 100 years old, or with a trunk diameter > 25 cm; their leaves are manufactured to high - quality, valuable ancient plants pu-erh tea (APPT). In this study, a fermentation of APPT were developed, and outstanding sweetness of APPT infusion was observed. During fermentation, the content of soluble sugars, theabrownins (*p* < 0.05), as well as 41 metabolites were increased [Variable importance in projection (VIP) > 1.0; *p* < 0.05 and Fold-change (FC) FC > 2]; While relative levels of 72 metabolites were decreased (VIP > 1.0, *p* < 0.05 and FC < 0.5. *Staphylococcus*, *Achromobacter*, *Sphingomonas*, *Thermomyces*, *Rasamsonia*, *Blastobotrys*, *Aspergillus* and *Cladosporium* were identified as dominant genera, and their relative levels were correlated with contents of characteristic components (*p* < 0.05). Together, changes in sensory characteristics, chemical composition and microbial succession during APPT fermentation were investigated, and advanced the formation mechanism of its unique quality.

## Highlights

• The soluble sugar content of the fermented ancient plants Pu-erh tea was 7.55%.
• Microbial succession during the fermentation was analyzed.
• The relative levels of 113 metabolites changed significantly during fermentation.
• The microbial genera responsible for compositional changes were identified.

## 1 Introduction

The tender leaves of the tea plant [*Camellia sinensis* var*. sinensis*, or var. *assamica* (L.) O. Kuntze] are used to produce tea, the world’s second most popular beverage after water (Wang et al., 2020). In 2021, the area of tea cultivation was 8,636,270 ha, spread over 48 countries (http://www.fao.org/faostat). In modern tea plantations, tea plants are generally pruned to a height of 70 to 80 cm to improve yields and harvesting efficiency, which dominates worldwide.

Yunnan Province in southwest China is a major area of tea plant cultivation; there are more than 460,000 ha of tea plantations, including for the famous pu-erh tea and black tea. There are many ancient tea plants in the cities of Yunnan Province, Xishuangbanna, Pu'er, and Lincang (Zheng et al., 2019, Zi et al., 2020). According to the Forestry Industry Standards of the People's Republic of China (LY/T 3311-2022), “ancienttea plants” are defined as tea trees > 100 years old, or with a trunk diameter > 25 cm (Lan et al., 2022). In ancient tea plantations, there is no modern cultivation management, for example, tea plants are not pruned, so they can grow to a height of several meters and receive little or no fertilizer; there are at least 220,000 ha ancient tea plantation in Yunnan province. The fresh leaves of ancient plants are manufactured to sun-dried green tea, and then fermented into high-quality, valuable pu-erh tea, named ancient plants pu-erh tea (APPT) (Ge et al., 2021). APPT possess thicker leaves with more leather substances, clearer veins; the flavor is deeper, thicker and could retain for a longer time; the taste is complex mixing rough, fine, rich and smoot; the aftertaste is sweetness (Mei, 2016). Due to the unique and desirable flavor, APPT commands a relatively high price compared with normal tea from young plants (Liang et al., 2006).

Pu-erh tea is a famous traditional Chinese tea, which classified as raw or ripen pu-erh tea manufactured by compression or microbial fermentation using sun-dried green tea leaves as raw material (Wang et al., 2018). Ripen pu-erh tea has unique sensory characteristics, including a brownish-red tea infusion, a mellow taste, and a stale flavor, which are distinct from other types of tea. In addition, it also has various health beneficial effects, such as anti-hyperlipidemia, anti-obesity, anti-oxidant, anti-tumor (Lee & Foo, 2013). Ripen pu-erh tea is popular in Southeast Asia and is becoming increasingly popular in Western countries. The natural microbial fermentation is an essential part of ripen pu-erh tea manufacture; sun-dried tea leaves are pile-fermented under conditions of high humidity and a temperature of 40–60 °C for a few weeks, which results in oxidation, degradation, and condensation of the chemical components of the leaves (Zhu et al., 2020). Both culture-dependent and independent methods showed the importance of microbial diversity and succession during fermentation, which strongly influence the sensory quality and chemical composition of tea leaves; the major microbial genera include *Aspergillus*, *Penicillium*, *Rhizopus*, and *Saccharomyces* (Ma et al., 2017). In a previous report, the microbial succession, association, activity and metabolite changes were determined during the fermentation of pu-erh tea using sun-dried green tea leaves from modern tea plants as raw material (Zhao et al., 2019).

Differences in raw material source and quality, and pile-fermentation processes also affect the composition and succession of fermenting microorganisms, which in turn, affects the quality of the resulting tea leaves (Li, Feng, Luo, Yao, Zhang, & Zhang, 2018). Therefore, fermentation of pu-erh tea using ancient plants sun-dried green tea as raw material may involve a microbiome distinct from other tea fermentations and result in distinct chemical changes. However, little is known about the metabolism of tea chemical compounds and microbial succession during APPT fermentation.

In this study, changes in sensory characteristics, metabolism of chemical compounds and microbial composition were investigated during APPT fermentation, by sensory evaluation, metabolomic analysis, and high-throughput sequencing. In addition, the correlation between the dominant microorganisms and changes in chemical composition was analyzed, to improve understanding of the relationships between them.

## 2 Materials and methods

### 2.1 Chemical standards

HPLC-grade acetonitrile and methanol were from Beijing Mirida Technology Co., Ltd., (Beijing, China). Reference standards (>98% purity) of the 18 characteristic tea compounds, i.e., (+)-catechin, gallic acid, (−)-epicatechin, (−)-epigallocatechin, (−)-epicatechin gallate, (−)-epigallocatechin-3-gallate, (−)-gallocatechin, (−)-catechin gallate, (−)-gallocatechin gallate, ellagic acid, caffeine, theophylline, myricetin, quercetin, luteolin, kaempferol, rutin, taxifolin, were from Manster Biotechnology Co., Ltd. (Chengdu, China).

### 2.2 Fermentation of pu-erh tea and sample collection

Fresh leaves with one bud and three leaves were plucked from “Ancient tea plants” at Laobanzhang Village (100°29′52.638''N, 21°43′43.6584''E) in Xishuangbanna Autonomous Region, Yunnan Province, China. Fresh leaves were spreading for 4–5 h, and fixed in a hotpan with about 280 °C for 30 min. Which were rolled, then sun-dried to moisture less than 12%, and this manufactured tea leaves were sun-dried green tea.

The fermentation of APPT was developed using the traditional method at Menghai Dazhuo Tea Co., Ltd., Yunnan, China, from June to July 2022. The raw materials, water, containers and the environment were not sterilized and no fermentation starter culture was used, so microorganisms from the tea leaves performed the fermentation. The tea leaves were sprayed with water to achieve a moisture content of ∼40%, then formed into piles ∼1 M high. The tea piles were turned over and re-formed into piles four times (Table S1), depending on the internal temperature, and the fermentations were terminated after 36 days. During each turning, 2 kg samples of fermenting tea leaves were collected and divided into two parts, one of which was freeze dried and subjected to sensory evaluation and compositional analysis. The other part was stored at −80 °C for high throughput sequencing analysis. All samples were analyzed by sensory evaluation, HPLC, spectrophotometry and metabarcoding analysis of microbial DNA. In addition, the raw material and samples from on tea pile were subjected to metabolomics analysis. Detailed sample information is provided in Table S1.

### 2.3 Sensory evaluation of tea leaves

The sensory evaluation of tea leaves was performed according the Chinese standard (Methodology for sensory evaluation of tea, 2018, Gong et al., 2018). The sensory evaluation was carried out in a purpose-built sensory review room at 25 °C and relative humidity < 70%. Briefly, each sample was evenly spread out on bamboo dividing trays and the appearance of these teas, including streaking, clarity, integrity and color were assessed visually and graded. Secondly, dry tea leaves (3 g) were added to boiling water (150 mL) for 5 min, then nine trained sensory testers (five males and four females, aged 24 to 48 years) assessed the infused tea for color, aroma and flavor. In addition, taste profiles (bitterness, astringency, thickness, sweetness, sourness and umami) were assessed using quantitative descriptive analysis (Fan et al., 2021). The intensity level of the flavor attributes was assessed using a scale ranging from 0 (undetectable) to 10 (very strong). The CIELAB color parameters were determined, i.e., L* - lightness, a* - redness (+) and greenness (−) and b* - yellowness (+) and blueness (−).

### 2.4 Analysis of characteristic compounds in tea leaves

The total contents of water extracts, tea polyphenols, soluble sugars, theaflavins, thearubigins, and theabrownins in tea infusions were determined as described previously (Wang, Peng, & Gong, 2011). The 18 characteristic tea compounds (Section 2.1) were determined by HPLC with an Agilent 1200 series HPLC system (Agilent Technologies, Santa Clara, CA, USA) and separated in an analytical Agilent Poroshell 120 EC-C 18 column (4.6 mm × 100 mm, 2.7 μm, Agilent Technologies, Santa Clara, CA, USA),as described previously (Nian et al., 2019).

### 2.5 Untargeted metabolomics analysis

#### 2.5.1 Metabolite extraction

Tea leaf samples (50 mg) in 2 mL Eppendorf tubes were treated with extraction solution (280 µL, methanol/water = 2:5 v/v) and 2-methylthiobenzothiazole (400 µL). After mixing, the samples were ground in a high-throughput tissue crusher (Wonbio-96c; Shanghai Wonbio Technology Co., Ltd., China) (6 min, −10 °C, 50 Hz). The samples were then extracted in a low-temperature ultrasonic bath (SBL-10TD, Ningbo Coenz Biotechnology Co., Ltd., China) for 30 min (5 °C, 40 kHz) and then left to stand at −20 °C for 30 min. The samples were centrifuged using a high-speed refrigerated centrifuge (Centrifuge 5430R, Eppendorf, Germany) (10 min, 4 °C, 13,000×*g*). An aliquot of supernatant (350 µL) was dried under nitrogen, then redissolved (100 µL, isopropanol/acetonitrile = 1:1 v/v). The solution was vortexed for 30 s, then sonicated for 5 min at 40 KHz in an ice-water bath. The solution was centrifuged for 10 min (13,000×*g*, 4 °C), then an aliquot of supernatant (20 µL) from each sample was mixed to generate a quality control (QC) sample and all the samples analyzed by UHPLC-MS/MS at Majorbio Bio-Pharm Technology Co., Ltd. (Shanghai, China).

#### 2.5.2 UHPLC-MS/MS analysis

Chromatographic separation of the samples was performed on a Thermo UHPLC Vanquish Horizon system, equipped with an Accucore C30 Column (100 mm × 2.1 mm i.d., 2.6 µm; Thermo Fisher) maintained at 40 °C. Separation of sample components was achieved at a 0.4 mL/min flow rate, with mobile phases: 10 mM ammonium acetate in 1:1 v/v acetonitrile/water (solvent A); 2 mM ammonium acetate and 1% v/v formic acid in 10:88:2 v/v/v acetonitrile/isopropanol/water (solvent B). The column was eluted with a linear gradient: 0 min, 35% B; 4 min, 60% B; 12 min, 85% B; 15 min, 100% B, held until 17 min; 18 min, 0% B, held until 20 min. The samples were maintained at 4 °C in the autosampler, before analysis.

The mass spectrometric data were acquired using a Thermo UHPLC-Q-Exactive HF-X Benchtop Orbitrap mass spectrometer, equipped with a heated-electrospray ionization (HESI) source, operating in both positive and negative ion mode. The analysis conditions were set as follows: Sheath gas flow rate 60 psi; aux gas flow rate 20 psi; aux gas heater temperature 370 °C; ion-spray voltage floating (ISVF) at −3000 V in negative mode and at +3000 V in positive mode; normalized collision energy, 20–40–60 V rolling for MS/MS. Data acquisition was performed in data dependent acquisition (DDA) mode, over a mass range of 200–2000 *m*/*z*.

#### 2.5.3 Data analysis

The pretreatment of LC/MS raw data was performed by Progenesis QI (Waters Corporation, Milford, USA) software, and a three-dimensional data matrix in CSV format was exported. The information in this three-dimensional matrix included: sample information, metabolite name and mass spectral response intensity. Internal standard peaks, as well as any known false positive peaks (including noise, column bleed, and derivatized reagent peaks), were removed from the data matrix, deredundant and peak pooled. At the same time, the metabolites were identified by searching database, and the main databases were the HMDB (https://www.hmdb.ca/), Metlin (https://metlin.scripps.edu/).

### 2.5 Microbial succession analysis

#### 2.6.1 DNA extraction and PCR amplification

Microbial genomic DNA was extracted from each tea leaf sample using the E.Z.N.A Mag-Bind Soil DNA extraction Kit (Omega Bio-tek, Norcross, GA), following the manufacturer’s instructions (Hu et al, 2021). The concentration and purity of extracted DNA were measured spectrophotometrically with a NanoDrop 2000 (Thermo Fisher Scientific, Waltham, MA) and the quality was checked by 1% agarose gel electrophoresis. The V3-V4 region of the bacterial 16S rRNA gene and the fungal internal transcribed spacer (ITS)1 DNA region were amplified using primers 338F (5′-ACTCCTACGGGAGGCAGCAG-3′) and 806R (5′-GGACTACHVGGGTWTCTAAT-3′) as well as ITS1F (CTTGGTCATTTAGAGGAAGTAA) and ITS2R (GCTGCGTTCTTCATCGATGC), respectively. The PCR reaction system consisted of 5× FastPfu buffer (4 μL), forward primer (5 μmol/L; 0.8 μL), 2.5 mM dNTPs (2 μL), reverse primer (5 μmol/L: 0.8 μL), FastPfu polymerase (0.4 μL), BSA (0.2 μL), template DNA (10 ng) and ddH_2_O (20 μL). The PCR (ABI GeneAmp PCR System 9700, Thermo Fisher) amplification procedure was as follows: 95 °C for 3 min, 27 cycles (95 °C for 30 s, 55 °C for 30 s, 72 °C for 30 s), followed by final extension at 72 °C for 10 min. Each sample was amplified three times in duplicate.

#### 2.6.2 Illumina MiSeq sequencing

The amplified PCR products were purified using an AxyPrep DNA Gel Extraction Kit (Axygen Biosciences, Union City, CA), eluted with Tris-HCl and separated by 2% agarose gel electrophoresis. The recovered products were quantified using QuantiFluor-ST (Promega, Madison, WI) at Majorbio Bio-Pharm Technology Co., Ltd. (Shanghai, China). After filtering the raw data, the data were processed and stitched together using Flash software (version 1.2.11, https://ccb.jhu.edu/software/FLASH/index.shtml). Operational taxonomic units (OTUs) clustering for species classification analysis was performed with Uparse software (v11, https://www.drive5.com/uparse/), based on the Silva (v138, https://www.arb-silva.de/) 16S rRNA database, Unite (8.0, https://unite.ut.ee/) ITS database, Qiime (v1.9.1) platform and RDP classifier (v2.13, https://sourceforge.net/projects/rdp-classifier/). Community richness indices (ACE and Chao indices), community diversity (Shannon and Simpson indices), and Good’s coverage of sequencing were assessed with Mothur Software (v1.30.2, https://www.mothur.org/wiki/Download_mothur). The sequencing data for the bacterial 16S rRNA genes and the fungal ITS genes are available at the Sequence Read Archive under project code (PRJNA1001624).

### 2.7 Statistical analysis

The experimental data are presented as the mean ± standard deviation (SD) from at least triplicate experiments for each condition. One-way analysis of variance (ANOVA) with Dunnett’s multiple comparisons test was used to distinguish the significance level of differences between samples; *p* < 0.05 was considered to be statistically significant. Heatmaps were plotted using the TBtools software (Toolbox for Biologists; Version 1.082, China). Linear discriminant analysis Effect Size (LEfSe) was performed using the Galaxy tool (https://huttenhower.sph.harvard.edu/galaxy/). The correlation between dominant genera and chemical composition was calculated by Spearman’s test and visualized by Python (version 3.6.6).

## 3 Results and discussion

### 3.1 Changes in sensory characteristics

**Fig. 1:**
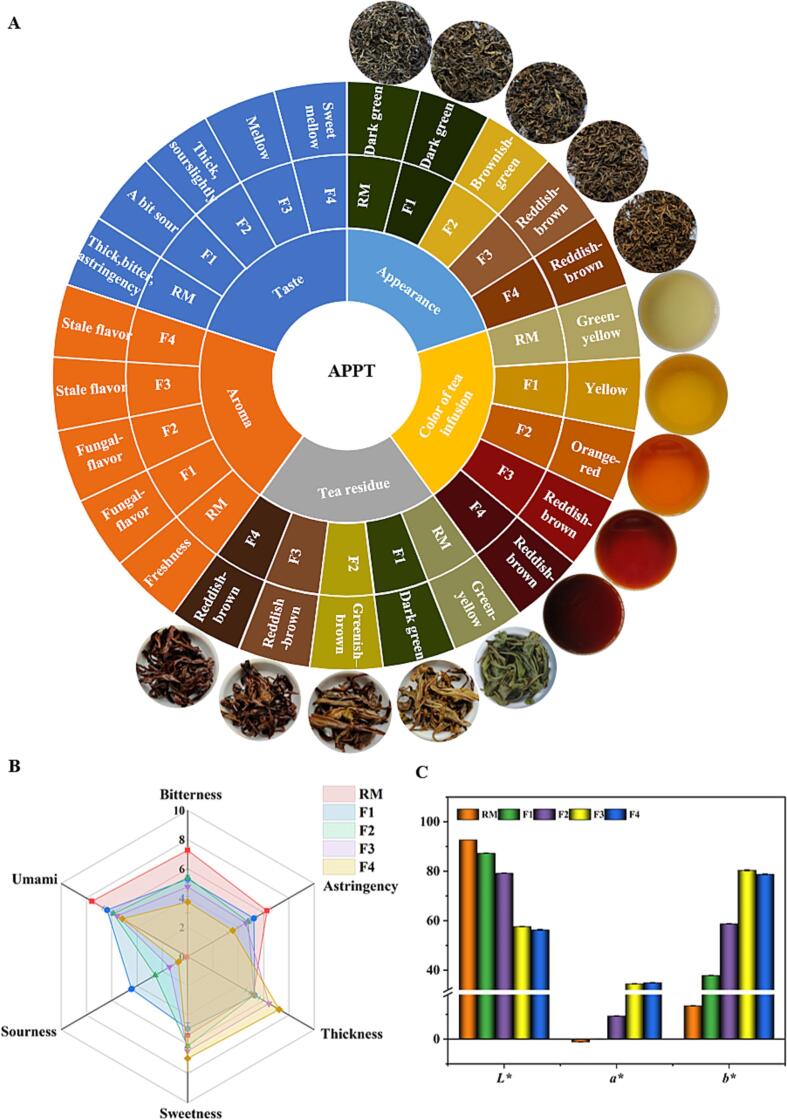
Sensory attribute wheel illustrating changes in sensory characteristics during tea fermentation (A); taste sensory scores of tea infusions (B); color index (C).

The infusion of the green tea raw material (RM) was yellow-green in color, bitter and thick in taste, with a fresh aroma. During the initial stage of fermentation (sample F1), the tea infusion was yellow with little sour taste and at the end of fermentation (sample F4), the tea infusion became reddish brown, mellow in taste, with a stale aroma (Fig. 1A). These changes in sensory attributes are similar to those generally observed in pu-erh tea fermentations (Zhao et al, 2019). Compared with the RM, the sweetness (6.97), thickness (7.23), sourness (0.73), a* (34.91) and b* (78.77), of sample F4 were higher (*p* < 0.05), whereas the bitterness (3.73), astringency (3.57), freshness (5.17) and L* (56.32), were lower (*p* < 0.05; Fig. 1B-C). Overall, during the fermentation, the astringency and bitterness decreased, the infusion turned from yellow to reddish-brown, a stale aroma formed and the resulting APPT had an outstanding sweetness score.

### 3.2 Changes in characteristic components

**Fig. 2:**
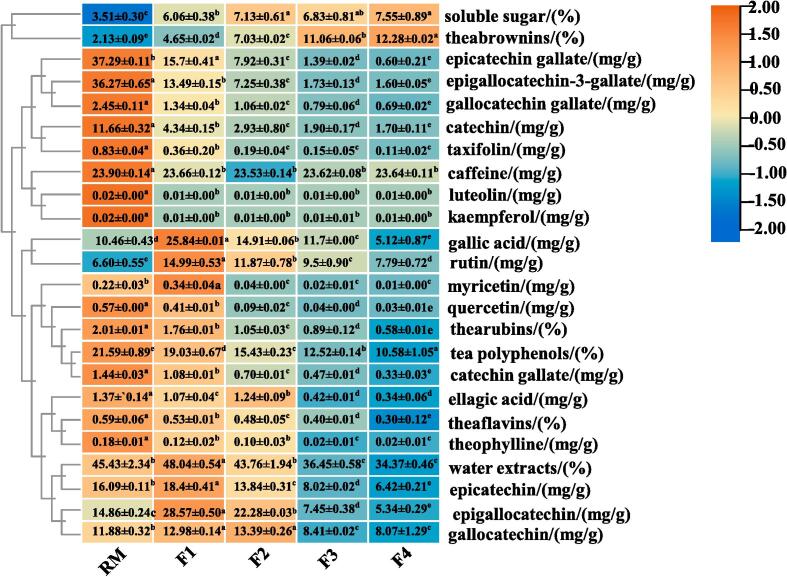
Content changes of 24 characteristic compounds and other compositional quality characteristics in tea leaves during fermentation.

It is well-established that changes in tea sensory characteristics are associated with changes in chemical composition. Therefore, 24 compositional characteristics, including water extracts, tea polyphenols, soluble sugars, three tea pigments, two phenolic acids, eight catechins, and six flavonoids were measured (Fig. 2). During fermentation, the contents of five catechins (epicatechin gallate, epigallocatechin-3-gallate, gallocatechin gallate, catechin, and catechin gallate), taxifolin, caffeine, luteolin, kaempferol, quercetin, thearubigins, tea polyphenols, theaflavins and theophylline, decreased (*p* < 0.05), whereas those of soluble sugars and theabrownins increased (*p* < 0.05).

Water extracts are an important indicator of the quality of tea leaves and contain the main flavoring substances in tea infusion (Li et al, 2017). The water extracts increased significantly in F1 and then decreased (Fig. 2), in agreement with a previous report (Gong, Zhou, Zhang, Song, & An, 2005), that the water extracts decreased during fermentation.

Tea polyphenols are the main components that contribute to the flavor and health benefits of tea. Tea polyphenols in the RM were 21.59% significantly decreased to 10.58% in F4 (Fig. 2), consistent with a previous report (Zhao et al, 2019). In addition, the contents of tea polyphenols in other dark teas (e.g., Pu-erh, Qingzhuan, and Fu brick) were reduced by microbial fermentation (*p* < 0.05) (Gong et al., 2005, Cheng et al., 2020, Qin et al., 2012). This probably results from the oxidation, condensation and polymerization of tea polyphenols, which produces large amounts of pigments or polymers, which bind to proteins (Zhu et al., 2020).

**Fig. 3:**
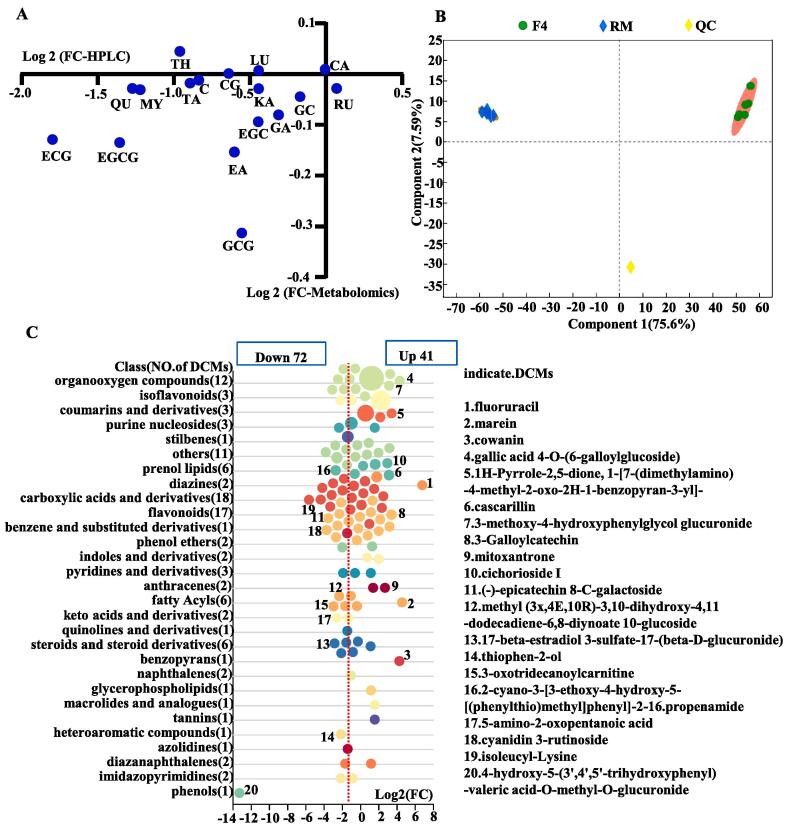
Changes in relative levels (RLs) of metabolites during pile fermentation of tea leaves. Change trend of 17 metabolites identified both by HPLC and metabolomics analysis (A); PCA analysis of peak areas (B); changes and classification of all 113 differential metabolites (C).

Soluble sugars reduce the bitterness of tea, from compounds such as theobromine and polyphenols, which are the main flavor compounds of tea (Hu et al., 2021). Notably, the soluble sugar content increased from 3.51% in RM to 7.55% in F4 (*p* < 0.05; Fig. 3), consistent with a similar trend in a previous report (Li et al., 2022). Interestingly, Xu et al (2022) suggested that the increase in soluble sugars after fermentation was due to the secreted cellulase and pectinase enzymes by the fungus that can degrade cellulose, pectin and other polysaccharides in pu-erh tea. The high soluble sugar content in the fermented tea leaves correlated with the outstanding sweetness sensory score of APPT infusion.

Theaflavins, thearubigins and theabrownins are the oxidation products of catechins, and are the main pigments responsible for the color of tea infusion. During fermentation, the content of both theaflavins and thearubigins decreased (*p* < 0.05), which contributed to the reduced bitterness and astringency of the tea infusion (Fig. 2). However, theabrownins, which are mainly responsible for the characteristic brown color of tea infusion, increased from 2.13% to 12.28%. Theabrownins have various health-beneficial effects, such as antioxidant, anti-hypercholesterolemic (Huang et al., 2019) and anti-tumor effects (Xu et al., 2020). Theabrownins are negatively associated with bitterness and astringency (Chen et al., 2022) and make a large contribution to the formation of the mellow flavor and distinctive color of tea (Cheng et al., 2020). Therefore, it appears that the continuous increase in theabrownin content in APPT during fermentation contributes to the unique quality characteristics of APPT. Consequently, theabrownins can be used as a marker of APPT maturity, enabling real-time monitoring of the fermentation process. In summary, the contents of catechins (epicatechin gallate, epigallocatechin-3-gallate, gallocatechin gallate, catechin, catechin gallate), taxifolin, luteolin, kaempferol, quercetin, thearubigins, tea polyphenols, theaflavins and theophylline decreased (*p* < 0.05), whereas those of soluble sugars and theabrownins increased (*p* < 0.05) during fermentation. Overall, these chemical compositional changes endowed APPT with a sweet, mellow taste and a reddish-brown infusion.

### 3.3 Metabolomic changes

Metabolomic analysis of RM and the final fermented tea leaves (sample F4) was performed to compare the chemical compositional changes during the fermentation. A total of 17 metabolites were identified in both HPLC and metabolomics analyses. Epicatechin gallate, epigallocatechin-3-gallate, catechin, gallocatechin gallate, epigallocatechin, gallocatechin, quercetin, myricetin, taxifolin, ellagic acid and kaempferol all decreased in F4, compared with RM (Fig. 3A, Table S2). Principal components analysis (PCA; Fig. 3B) resulted in a wide separation between the closely grouped replicates of RM and F4, indicating that pile-fermentation of the tea leaves produced marked changes in the metabolite composition. After fermentation, the relative levels (RLs) of 72 metabolites decreased compared with RM (VIP > 1, *p* < 0.05 and FC < 0.5), e.g., fluoruracil, marein and gallic acid 4-O-(6-galloylglucoside). These decreased metabolites were grouped into carboxylic acids and derivatives (16 metabolites), flavonoids (10 metabolites), organooxygen compounds (6 metabolites), fatty acyl compounds (5 metabolites), steroids and steroid derivatives (5 metabolites), prenol lipids (3 metabolites), isoflavonoids (2 metabolites), and keto acids and derivatives (2 metabolites) (Fig. 3C). The RLs of 41 metabolites increased after fermentation (VIP > 1, *p* < 0.05 and FC > 2), e.g., (−)-epicatechin 8-C-galactoside, cyanidin 3-rutinoside and isoleucyl-lysine. These were grouped into flavonoids (7 metabolites), organooxygen compounds (6 metabolites), coumarins and derivatives (3 metabolites), prenol lipids (3 metabolites), anthracenes (2 metabolites), carboxylic acids and derivatives (2 metabolites), diazines (2 metabolites), indoles and derivatives (2 metabolites) (Fig. 3C). In summary, active metabolism was observed for carboxylic acids and derivatives, flavonoids, organooxygen compounds, anthracenes, fatty acyls, prenol lipids, steroids and steroid derivatives, coumarins and derivatives, and isoflavonoids, which is consistent with changes observed in industrial pu-erh tea fermentation (Zhao et al., 2019).

### 3.4 Changes in microbial diversity during fermentation

A total of 632,598 valid bacterial sequences was identified, then clustered into 332 bacterial OTUs, assigned to 10 phyla, 16 classes, 56 orders, 100 families, 181 genera, and 221 species of bacteria. A total of 842,926 valid fungal sequences was identified and clustered into 436 fungal OTUs, assigned to 5 phyla, 22 orders, 65 families, 131 families, 198 genera, and 249 species of fungi.

#### 3.4.1 Alpha diversity

**Fig. 4:**
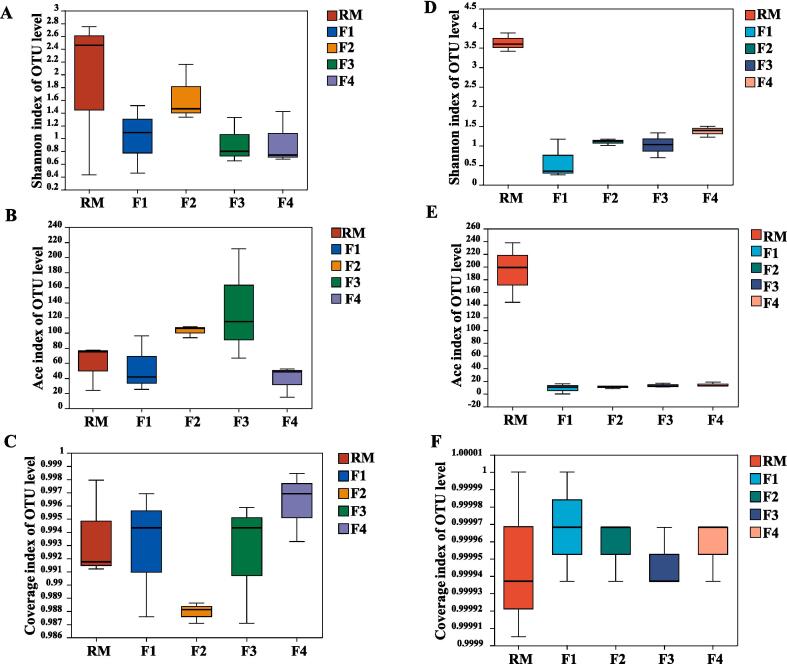
Alpha diversity indices (Shannon, ACE and coverage) of bacterial (A–C) and fungal (D–F) microbiomes of tea leaves in pile-fermentation. RM: raw material (sun dried green tea leaves); F1–F4, timed samples collected during fermentation (Table S1).

Alpha diversity is the observed richness (number of taxa), or evenness (the relative abundances of those taxa) of a microbiome. The microbial diversity and richness/abundance of the tea leaf microbiome were assessed using the Shannon and ACE indices, respectively (Fig. 4). All the sample coverage indices were greater than 0.980 (Fig. 4C, F), indicating that the sequencing depth was sufficient and accurately reflects the microbiome composition (Shi et al., 2021). The bacterial diversity (Shannon index) initially decreased markedly (RM to F1), then stayed constant apart from a small increase in F2 (Fig. 4A). The bacterial abundance (ACE index) gradually increased from RM to F3, then decreased markedly (Fig. 4E). The fungal diversity decreased markedly from RM to F1, then gradually increased, whereas the fungal abundance also decreased markedly from RM to F1, then remained relatively constant (Fig. 4D, E). The high ACE index/fungal abundance in the RM (Fig. 4E), is consistent with the industrial fermentation of pu-erh tea and indicates that the initially very abundant fungi in RM cannot adapt to the hot and humid fermentation environment (Li et al., 2022).

#### 3.4.2 Beta diversity

**Supplementary Figure 1:**
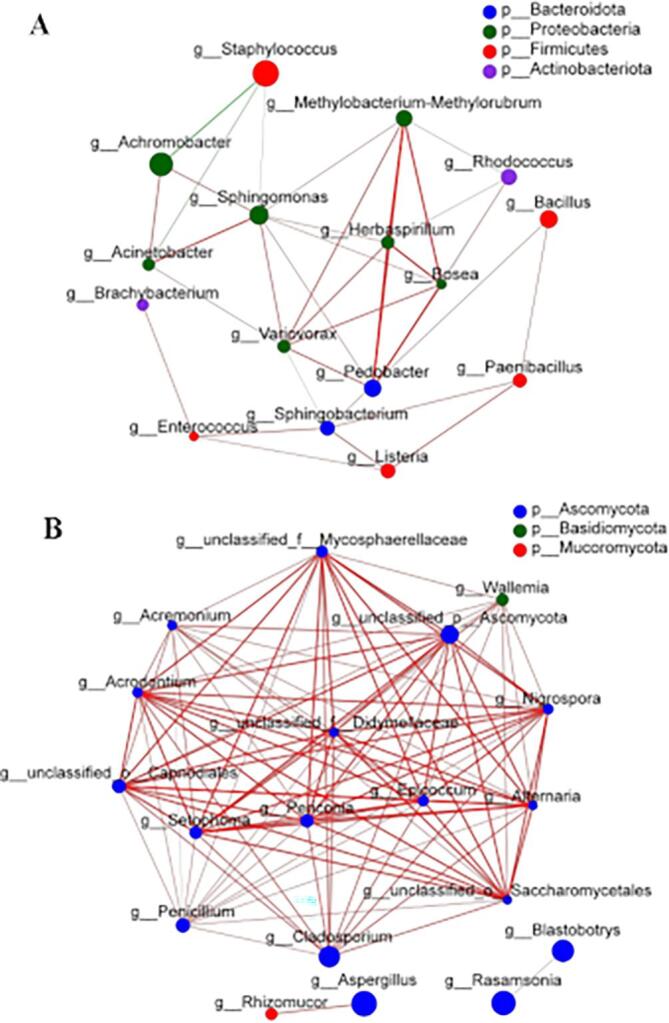


Beta-diversity is the variability in microbial composition (the identity of taxa observed) in a microbiome. Principal components analysis (PCA) was performed on the OTU data, based on the Bray-Curtis distance matrix. PC1 and PC2 together explained 72.76% of the variation in bacterial Beta diversity and 69.62% of the variation in fungal Beta diversity (Fig. S1A, B), indicating that PCA is a good reflection of the factors influencing the differences in bacterial and fungal composition among different tea samples. It is notable that both bacterial and fungal microbiomes can be classified into three groups, i.e., group I (RM), group II (F1), and group III (F2, F3 and F4; Fig. S1A, B). In summary, the microbiome structure changed markedly in the early stage of fermentation, then stabilized in the middle and late stages.

### 3.5 Microbial succession during fermentation

#### 3.5.1 Bacterial composition

**Fig. 5:**
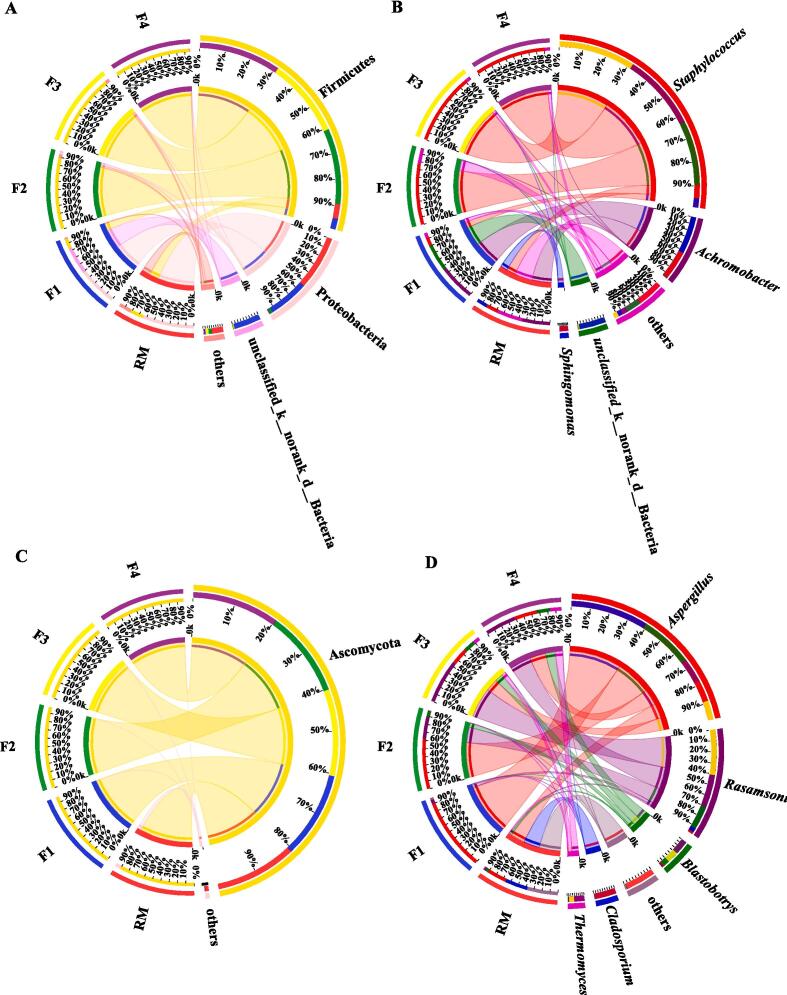
Relative abundance of bacteria at (A) phylum and (B) genus levels and relative abundance of fungi at (C) phylum and (D) genus levels. (Note: Taxonomic abundances < 1% were classified as “others”).

To investigate microbial succession during APPT fermentation, the compositional variation of bacteria and fungi at the phylum and genus levels were analyzed. The bacterial microbiome of all samples included 10 phyla, with three dominant (relative abundance > 1%), phyla, namely Firmicutes, Proteobacteria, and unclassified_k_norank_d_Bacteria (Fig. 5A). Proteobacteria were predominant in RM with a relative abundance of 66.30%, which decreased to 49.73% in F1, 3.28% in F2, 0.36% in F3 and 0.76% in F4. The relative abundance of Firmicutes increased significantly from 17.95% (RM) to 91.43% (F2), then plateaued at 95.00% in F3, and 98.36% in F4 (Fig. 5A). Similarly, the dominant bacteria in pile fermentation of tea leaves were Firmicutes (37.01%), Actinobacteriota (43.16%), and Proteobacteria (13.89%) (Zhang, Skaar, Sulyok, Liu, Rao, & Taylor, 2017). Proteobacteria was the dominant phylum in the early stage of tea fermentation, whereas Firmicutes was dominant (>90%) in the middle and late fermentation stages (Li et al., 2017).

At the genus level, four dominant genera (relative abundance > 1%) were identified as *Staphylococcus, Achromobacter*, unclassified_k_norank_d_Bacteria, and *Sphingomonas* (Fig. 5B). The major genera in RM were *Achromobacter*, *Staphylococcus*, and *Sphingomonas* with relative abundances of 36.63, 16.80 and 13.45%, respectively. *Staphylococcus* was the dominant genus in the middle and late stages of fermentation (F2, F3, F4) with an average relative abundance of 88.93%. Similarly, *Staphylococcus* multiplied markedly to 82.97% in the later stages of fermentation (Li et al., 2022). Further research is needed to fully elucidate the relative contributions of different phyla and genera to tea fermentation.

#### 3.5.2 Fungal composition

Ascomycota was the strongly dominant phylum throughout the fermentation; its relative abundance increased from 93.32 to 99.86% and plateaued in the middle of fermentation (Fig. 5C), consistent with a previous report (Zhang et al., 2017). At the genus level (Fig. 5D), five dominant fungal genera (relative abundance > 1%) were detected, including *Aspergillus*, *Rasamsonia*, *Blastobotrys*, *Cladosporium*, and *Thermomyces*. Of these, *Aspergillus* is considered to be the most abundant and dominant genus in microbial fermentation and commercially ripen pu-erh tea products (Zhu et al., 2020). The relative abundance of *Aspergillus* increased to 92.85% in F1, then decreased to 63.47% (F2), 22.28% (F3), 29.12% (F4). Similarly, the dominant microorganism at the genus level was *Aspergillus* (42.10%) in the early stage of pu-erh tea fermentation; its relative abundance decreased substantially to 6.51% after fermentation (Zhang, Zhang, Zhou, Ling, & Wan, 2013). In summary, the dominant fungi in young plant pu-erh tea fermentation are similar to those found in APPT fermentation, in this study. It appears that there is a relationship between the chemical composition and nutrient resources in the fermented tea and differences in microbial composition between raw and fermented tea (Li et al., 2022). Different raw materials and fermentation environments also appear to influence differences in the microbial composition in normal pu-erh tea fermentation (Li et al., 2018). The microbial diversity of the final product is related to the raw material, fermentation environment, and storage conditions. This study found *Achromobacter* and *Sphingomonas* to be dominant microorganisms for the first time in ripen pu-er tea, providing new possibilities for process modification and improvement.

#### 3.5.3 Biomarkers of microbial succession during fermentation

Differences in bacterial and fungal communities were analyzed using Linear discriminant analysis Effect Size (LEfSe), with a linear discriminant analysis (LDA) threshold of 2.0 (Fig. S2), to identify biomarkers of microbial succession and relative abundance changes between the different stages of fermentation. These biomarkers can be used for process monitoring of microbial succession during APPT fermentation. Two bacterial phyla and four bacterial genera were identified as biomarkers (LDA > 2, *p* < 0.05, Fig. S2A). At the phylum level, unclassified_k__norank_d__Bacteria and Firmicutes were biomarkers at the F1 and F2 stages, respectively. *Achromobacter*, *Sphingobacterium* and *Staphylococcus* were biomarkers for the early (F1), middle (F2) and late (F3) stages of fermentation, respectively, and *Achromobacter*, *Sphingobacterium*, and *Staphylococcus* were biomarkers in the final fermented tea leaves (F4), in agreement with previous reports on normal pu-erh tea fermentation (Ma et al., 2017, Zhao et al., 2019, Abe et al., 2008, Li et al., 2018).

Six fungi were identified as biomarkers at the genus level (LDA > 2, *p* < 0.05, Fig. S2B) *Penicillium* and *Wallemia* were biomarkers in RM, *Aspergillus* and *Rasamsonia* in F1 and F3, and *Blastobotrys* and *Thermomyces* in F4. In summary, *Aspergillus, Rasamsonia*, *Blastobotrys* and *Thermomyces* were biomarkers in fermented tea leaves, in agreement with previous reports of normal pu-erh tea fermentation (Ma et al., 2017, Zhao et al., 2019, Abe et al., 2008, Li et al., 2018).

#### 3.5.4 Microbial co-occurrence networks

The microbiome composition changed markedly during the fermentation and would be expected to strongly influence the chemical composition of the tea. To investigate the microbial interactions and growth-regulatory mechanisms during the fermentation, a co-occurrence network diagram of the top 20 genera in relative abundance at the genus level was constructed, with *p* > 0.05 and *|R|* < 0.6, separately for bacteria and fungi (Fig. S3) (Li et al., 2016).

The co-occurrence network diagram at the bacterial genus level (Fig. S3A) had 16 hubs and 70 linkages, of which 94.29% were positive and 5.71% were positively correlated. *Staphylococcus*, *Achromobacter*, *Sphingomonas*, and *Acinetobacter* negatively correlated (*p* < 0.05); it is noteworthy that the relative abundance of three of these genera decreased as the fermentation progressed, except for *Staphylococcus*. *Staphylococcus* had an average relative abundance of 88.93% in the middle and late stages of fermentation (F2, F3 and F4), and was the dominant genus during these stages. The *Variovorax* and *Sphingobacterium* genera were negatively correlated with each other (*p* < 0.05), and the other genera were positively correlated*.* The co-occurrence network diagram at the fungal genus level had 19 hubs and 238 linkages, all of which were positive, i.e., all the genera were significantly, positively correlated with each other (Fig. S3B). In summary, the growth-regulatory mechanisms of most of the microbial genera in the APPT fermentation significantly positively correlated.

### 3.6 Correlation between dominant genera and chemical composition

**Fig. 6:**
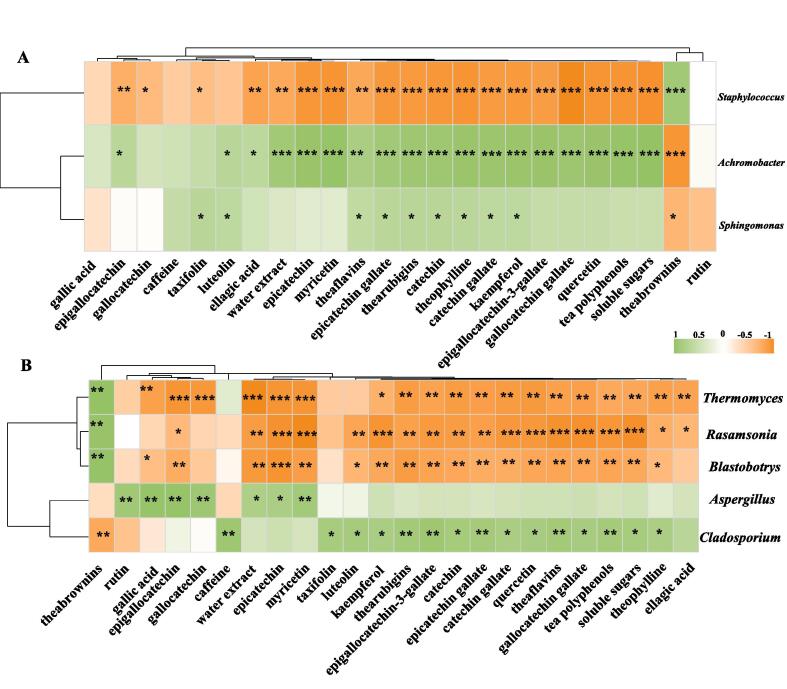
Heatmap of correlation analysis between characteristic tea compositional factors and dominant bacterial (A) and fungal (B) genera, using Spearman’s correlation coefficients. Each column in the graph represents a characteristic tea compositional factor, each row represents a genus, and each square represents the Spearman’s correlation coefficient between a genus and a metabolite. *p* < 0.05 is marked with *. Positive values (green) indicate a positive correlation and negative values (orange), a negative correlation. (For interpretation of the references to color in this figure legend, the reader is referred to the web version of this article.)

The formation of the characteristic chemical compositions of fermented foods is closely related to the microbial composition of their fermentation processes (Ferrocino et al., 2017). The main objective of this study was to investigate this relationship during the fermentation of APPT), to facilitate process and tea quality improvement; this relationship was assessed by Spearman’s correlation analysis (Fig. 6). All eight dominant genera correlated significantly with the characteristic components (*|r|* ≥ 0.7, *P* < 0.05) (Fig. 6A). The correlation between *Staphylococcus, Achromobacrer* and the characteristic components reached 80%. *Staphylococcus* was positively correlated with theabrownins and negatively correlated with most of the characteristic components. *Achromobacrer* showed a negative correlation with theabrownins and a positive correlation (*p* < 0.05) with luteolin, ellagic acid, water extracts, myricetin, theophylline, thearubigins, theaflavins, kaempferol, quercetin, polyphenols, soluble sugars and seven catechins (epigallocatechin, epicatechin, epigallocatechin, catechin, catechin gallate, epigallocatechin-3-gallate, gallocatechin gallate). *Sphingomonas* was negatively correlated (*p* < 0.05) with theabrownins, and positively correlated (*p* < 0.05) with taxifolin, luteolin, theophylline, epicatechin gallate, thearubigins, catechin, theaflavins, catechin gallate and kaempferol.

Fungi are mainly involved in forming the characteristic qualities of ancient plants pu-erh tea by secreting abundant extracellular enzymes, which catalyze various biochemical reactions. *Thermomyces*, *Rasamsonia* and *Blastobotrys* were positively correlated (*p* < 0.05) with theabrownins and negatively correlated (*p* < 0.05) with most of the characteristic components (Fig. 6B). *Cladosporium* was negatively correlated with theabrownins and positively correlated with caffeine, taxifolin, luteolin, kaempferol, thearubigins, quercetin, theaflavins, polyphenols, soluble sugars, theophylline, and four catechins (epigallocatechin-3-gallate, catechin, epicatechin gallate, catechin gallate) (*p* < 0.05). Notably, *Aspergillus* had the maximum relative abundance throughout the fermentation, but only showed significant positive correlations with rutin, gallic acid, epigallocatechin, gallocatechin, water extracts, epicatechin, and myricetin (*p* < 0.05). The metabolites produced by *Aspergillus* may be used by other microorganisms (Huang et al., 2022). During dark tea fermentation, *Aspergillus* produces CAZymes, which contribute to the mellow taste of tea infusion (Ma et al., 2021). *Aspergillus* can also secrete glycoside hydrolase, glycosyltransferases and vanillyl-alcohol oxidases during fermentation to catalyze the hydrolysis, oxidization, transformation, and biodegradation of phenolic compounds, thus decreasing the content of tea polyphenols and increasing the content of theabrownins and gallic acid (Liu et al., 2020).

This study clarified the influence of eight dominant genera on 24 characteristic components. Pile fermentation is responsible for the changes the sensory characteristics and composition, which contribute to the high quality of APPT. The interactions between the dominant microorganisms and the chemical composition strongly influences the sensory quality of APPT, reducing its astringency and bitterness, forming a reddish-brown color, as well as the stale aroma. However, the correlation analysis revealed only the phylum and genus level relationships between microorganisms and chemical composition. Therefore, future research should include isolation and characterization of the key microbial species and determine their individual contributions to the chemical composition of APPT.

## 4 Conclusion

This study investigated changes in sensory characteristics, metabolism of chemical compounds and microbial succession during the fermentation of ancient plants pu-erh tea (APPT). After fermentation, the astringency and bitterness of the fermented tea leaves were reduced and the soluble sugar content increased, resulting in an outstandingly sweet tea infusion. The relative levels of 113 chemical components changed significantly. *Staphylococcus*, *Achromobacter*, *Sphingomonas*, *Thermomyces*, *Rasamsonia*, *Blastobotrys*, *Aspergillus* and *Cladosporium* were the main microbial genera in the fermenting tea leaves; their relative abundances (RLs) and changes in their RLs correlated with changes in chemical composition.

## Ethical statement and sensory consent

The study involved sensory evaluation by trained or naive panelists. The age of the panel participants involved in the sensory evaluation ranged from 24 to 48 years. All members volunteered to participate in the sensory evaluation and agreed to its publication. In addition, appropriate protocols were used to protect the rights and privacy of all participants during the execution of the research.

## CRediT authorship contribution statement

**Teng Wang:** Methodology, Formal analysis, Investigation, Data curation, Writing – original draft. **Yan Ma:** Supervision, Funding acquisition, Project administration, Resources. **Ming Zhao:** Supervision, Funding acquisition, Project administration, Resources, Writing – review & editing.

## Declaration of Competing Interest

The authors declare that they have no known competing financial interests or personal relationships that could have appeared to influence the work reported in this paper.

## Data Availability

The authors are unable or have chosen not to specify which data has been used.
