# Supplementary figures and images for: Changes in sensory characteristics, chemical composition and microbial succession during fermentation of ancient plants Pu-erh tea
Source: Food Chem X. 2023 Nov 23;20:101003. doi: 10.1016/j.fochx.2023.101003 (PMC10739768; doi:10.1016/j.fochx.2023.101003)

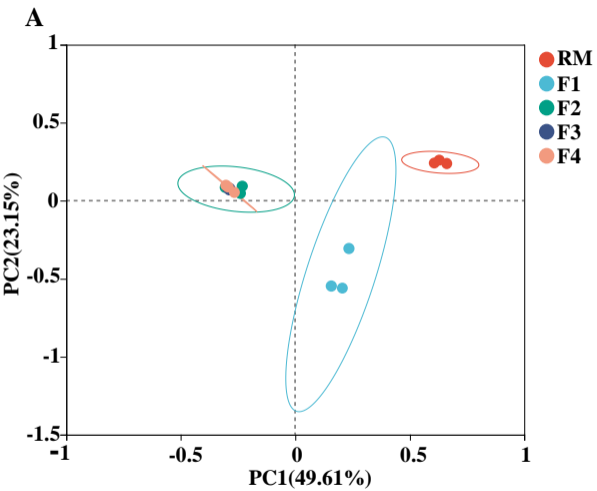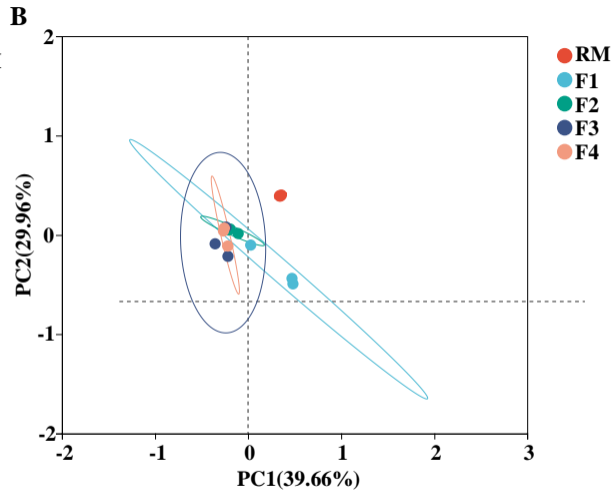

Supplement: Supplementary data 1 [file mmc1.pdf]

A

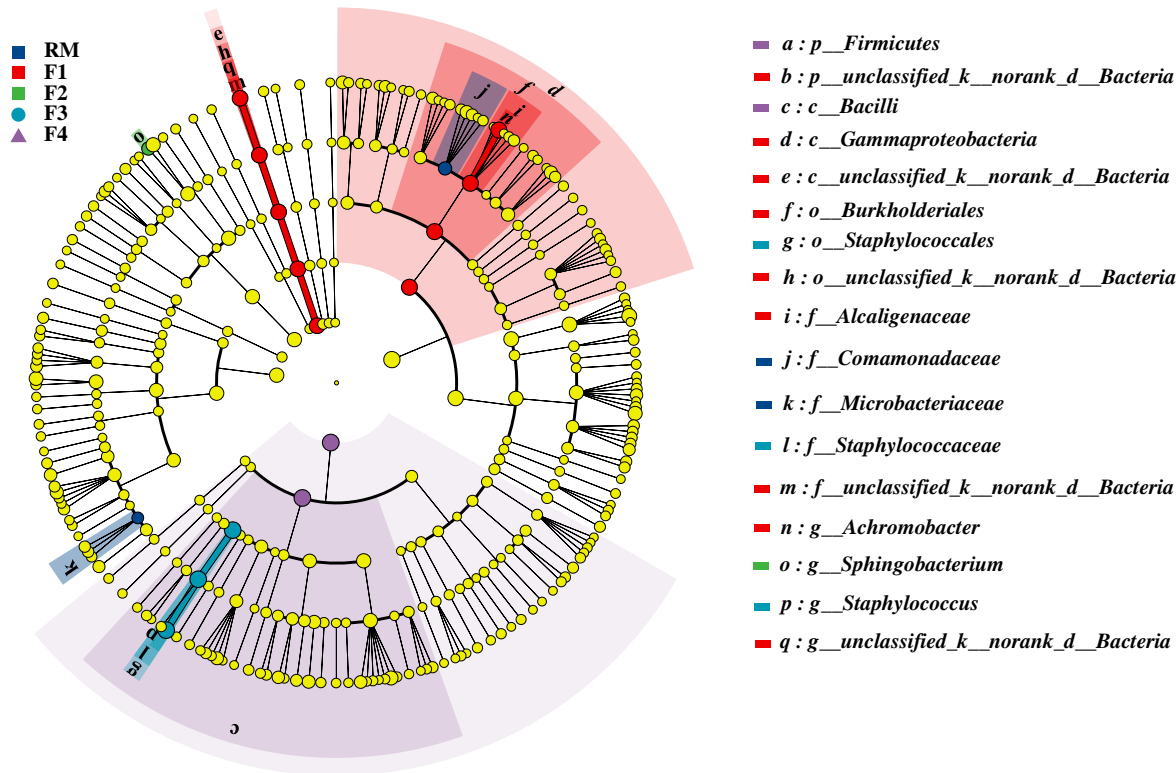

B

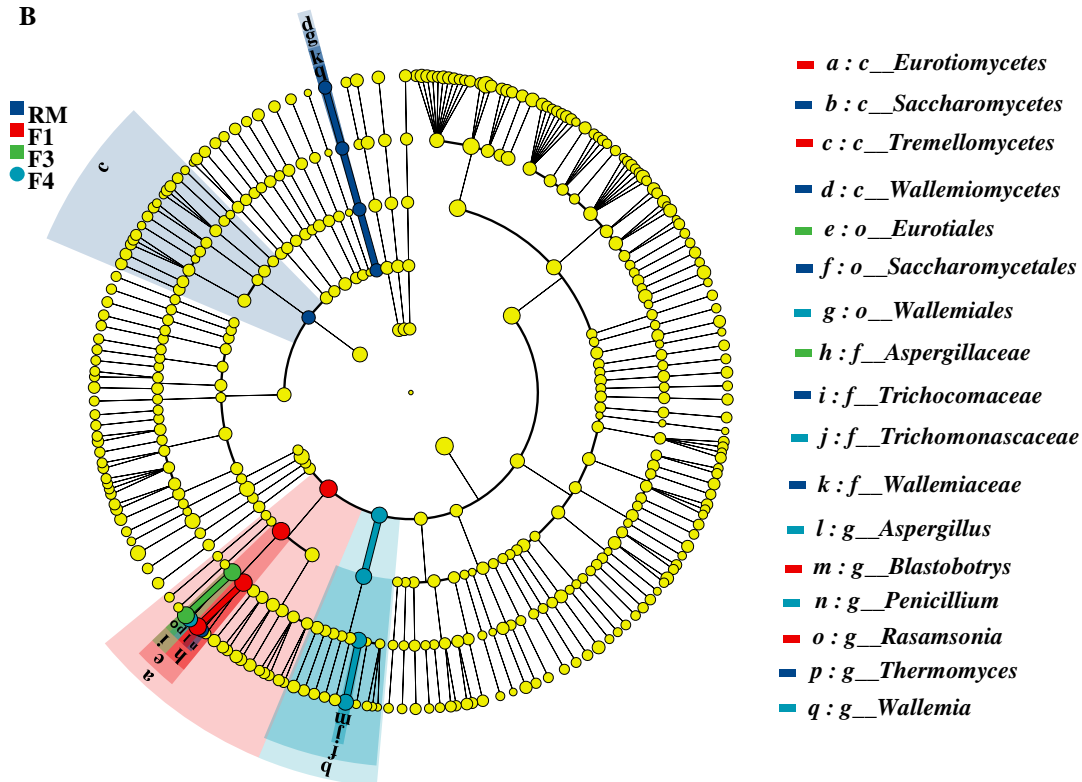

Supplement: Supplementary data 2 [file mmc2.pdf]
