# Supplementary material for: Changes in sensory characteristics, chemical composition and microbial succession during fermentation of ancient plants Pu-erh tea
Source: Food Chem X. 2023 Nov 23;20:101003. doi: 10.1016/j.fochx.2023.101003 (PMC10739768; doi:10.1016/j.fochx.2023.101003)
Supplement: Supplementary data 3 [file mmc3.docx]

Table S1. Samples of tea leaves collected in fermentation

| Sample | Collection date,  Day/mo (2022) | Analysis |
| --- | --- | --- |
| Raw material (RM) | 4/6 | sensory evaluation, measurement of components, metabarcoding metabolomics |
| First pile fermentation (F1) | 16/6 | sensory evaluation, measurement of components, metabarcoding |
| Second pile fermentation (F2) | 24/6 | sensory evaluation, measurement of components, metabarcoding |
| Third pile fermentation (F3) | 30/6 | sensory evaluation, measurement of components, metabarcoding |
| Fourth pile fermentation (F4) | 10/7 | sensory evaluation, measurement of components, metabarcoding analysis, metabolomics |
