# Supplementary material for: Changes in sensory characteristics, chemical composition and microbial succession during fermentation of ancient plants Pu-erh tea
Source: Food Chem X. 2023 Nov 23;20:101003. doi: 10.1016/j.fochx.2023.101003 (PMC10739768; doi:10.1016/j.fochx.2023.101003)
Supplement: Supplementary data 4 [file mmc4.docx]

Table S2. Differentially changed metabolites of RM and F4.

| ID | Metabolite | M/Z | Retention time | VIP_pred_OPLS-DA | FC(F4/RM) | Lo2(FC) | MEAN RM | MEAN F4 |
| --- | --- | --- | --- | --- | --- | --- | --- | --- |
| 1 | Fluoruracil | 148.053 | 3.640 | 2.585 | 109.939 | 6.781 | 0.04±0.00 | 4.89±0.23 |
| 2 | Marein | 433.115 | 3.952 | 2.576 | 23.487 | 4.554 | 0.22±0.36 | 5.10±0.57 |
| 3 | Cowanin | 443.220 | 4.596 | 2.542 | 19.230 | 4.265 | 0.26±0.01 | 5.00±0.58 |
| 4 | Gallic acid 4-O-(6-galloylglucoside) | 519.050 | 3.952 | 2.348 | 12.232 | 3.613 | 0.47±1.03 | 5.70±0.34 |
| 5 | 1H-Pyrrole-2,5-dione, 1-[7-(dimethylamino)-4-methyl-2-oxo-2H-1-benzopyran-3-yl]- | 299.100 | 2.001 | 2.874 | 6.961 | 2.799 | 1.03±1.21 | 7.20±0.30 |
| 6 | Cascarillin | 447.179 | 4.209 | 2.726 | 6.072 | 2.602 | 1.10±1.07 | 6.65±0.25 |
| 7 | 3-Methoxy-4-hydroxyphenylglycol glucuronide | 325.094 | 1.865 | 2.549 | 5.205 | 2.380 | 1.12±0.12 | 5.85±0.36 |
| 8 | 3-Galloylcatechin | 465.082 | 4.865 | 2.617 | 4.958 | 2.310 | 1.28±0.86 | 6.35±0.09 |
| 9 | Mitoxantrone | 489.174 | 3.012 | 2.600 | 4.698 | 2.232 | 1.32±0.01 | 6.22±0.22 |
| 10 | Cichorioside I | 477.120 | 4.389 | 2.038 | 4.433 | 2.148 | 1.18±1.15 | 5.24±0.32 |
| 11 | (+)-cis-Khellactone | 261.074 | 5.015 | 1.900 | 3.699 | 1.887 | 1.29±0.88 | 4.76±0.26 |
| 12 | Sennoside A | 907.186 | 4.733 | 2.176 | 3.555 | 1.830 | 1.74±0.83 | 6.18±0.19 |
| 13 | Citreoviridinol A1 | 403.177 | 5.780 | 2.190 | 3.530 | 1.820 | 1.42±0.32 | 5.00±0.60 |
| 14 | Ginkgolide B | 405.115 | 6.085 | 1.912 | 3.302 | 1.723 | 1.59±1.21 | 5.25±0.40 |
| 15 | Luteolin 7-malonylglucoside | 535.111 | 5.620 | 2.338 | 3.124 | 1.643 | 2.00±1.25 | 6.24±0.44 |
| 16 | N2,N2-Dimethylguanosine | 334.115 | 3.107 | 2.140 | 3.094 | 1.629 | 1.59±0.15 | 4.92±0.21 |
| 17 | Quercetin 3-(6''-acetylglucoside) | 507.116 | 4.454 | 2.465 | 2.983 | 1.577 | 2.22±0.01 | 6.62±0.23 |
| 18 | PL | 525.307 | 6.076 | 2.483 | 2.972 | 1.571 | 2.28±0.40 | 6.77±0.23 |
| 19 | Gallic acid 3-O-(6-galloylglucoside) | 519.050 | 3.787 | 2.015 | 2.952 | 1.562 | 1.91±0.14 | 5.65±0.33 |
| 20 | Brassicanal B | 297.066 | 2.639 | 2.183 | 2.766 | 1.468 | 2.01±0.69 | 5.57±0.31 |
| 21 | Doxorubicin-semiquinone | 582.177 | 5.475 | 1.959 | 2.647 | 1.404 | 2.19±0.74 | 5.8±0.20 |
| 22 | Maclurin 3-C-(6''-p-hydroxybenzoyl-glucoside) | 589.114 | 4.249 | 2.070 | 2.617 | 1.388 | 2.42±0.02 | 6.33±0.11 |
| 23 | Hydroxydesmethylpiperine sulfate | 392.080 | 4.413 | 1.832 | 2.577 | 1.365 | 1.96±0.09 | 5.05±0.29 |
| 24 | H-Asp(OBzl)-OH | 447.179 | 3.920 | 2.179 | 2.544 | 1.347 | 2.26±0.3 | 5.74±0.33 |
| 25 | Prolylglycine | 408.182 | 4.022 | 2.127 | 2.537 | 1.343 | 2.20±0.69 | 5.59±0.11 |
| 26 | Homopisatin | 318.136 | 3.203 | 1.761 | 2.483 | 1.312 | 1.98±1.83 | 4.91±0.60 |
| 27 | 3h-Serotonin | 221.067 | 2.814 | 1.992 | 2.446 | 1.290 | 1.99±0.12 | 4.87±0.17 |
| 28 | Myricetin 7-(6''-galloylglucoside) | 650.139 | 3.322 | 2.330 | 2.364 | 1.241 | 2.90±0.39 | 6.85±0.17 |
| 29 | Gallocatechin 3'-gallate | 479.055 | 4.835 | 1.946 | 2.323 | 1.216 | 2.74±0.85 | 6.36±0.10 |
| 30 | Tetramethylscutellarein | 377.082 | 2.636 | 1.818 | 2.310 | 1.208 | 2.37±0.54 | 5.49±0.31 |
| 31 | NNAL-N-glucuronide | 385.147 | 6.101 | 1.547 | 2.291 | 1.196 | 2.37±2.25 | 5.43±0.47 |
| 32 | Deoxycholylasparagine | 551.338 | 6.140 | 1.744 | 2.252 | 1.171 | 2.31±0.02 | 5.21±0.65 |
| 33 | Hydroxyclomipramine | 375.119 | 1.658 | 2.109 | 2.242 | 1.164 | 2.62±0.01 | 5.87±0.35 |
| 34 | Talviraline | 361.066 | 4.468 | 1.691 | 2.235 | 1.160 | 2.17±0.32 | 4.84±0.35 |
| 35 | Silmitasertib | 394.059 | 3.537 | 1.906 | 2.226 | 1.155 | 2.71±0.02 | 6.03±0.15 |
| 36 | Alvelestat | 528.132 | 5.326 | 2.069 | 2.195 | 1.134 | 2.61±0.01 | 5.73±0.29 |
| 37 | Prednisolone butyrate | 491.205 | 4.336 | 2.184 | 2.168 | 1.116 | 2.99±0.42 | 6.48±0.09 |
| 38 | 7,7,8,8-Tetrakis(methoxydiazenyl)bicyclo[4.2.0]octa-1(6),3-diene-2,5-dione | 387.076 | 2.385 | 1.806 | 2.094 | 1.066 | 2.78±0.38 | 5.82±0.34 |
| 39 | Gabazine | 619.251 | 6.524 | 1.665 | 2.083 | 1.058 | 2.57±0.47 | 5.35±0.86 |
| 40 | (-)-Gallocatechin gallate | 493.050 | 5.491 | 1.742 | 2.057 | 1.041 | 2.68±0.07 | 5.51±0.50 |
| 41 | 7-(alpha-D-Glucopyranosyloxy)-4-methyl-2H-1-benzopyran-2-one | 371.134 | 5.524 | 1.805 | 2.042 | 1.030 | 2.82±1.67 | 5.77±0.34 |
| 42 | N-alpha-acetyl lysine methyl ester | 405.272 | 6.430 | 1.729 | 0.500 | -1.001 | 5.48±0.18 | 2.74±0.04 |
| 43 | Peonidin 3-(6''-acetyl-galactoside) | 550.130 | 5.890 | 1.766 | 0.499 | -1.004 | 5.76±0.13 | 2.87±0.37 |
| 44 | L-4-Hydroxyglutamine | 388.141 | 4.533 | 2.050 | 0.499 | -1.004 | 6.48±0.06 | 3.23±0.95 |
| 45 | 5,6-Dihydro-1,10-phenanthroline | 365.173 | 6.004 | 1.977 | 0.494 | -1.019 | 5.58±0.07 | 2.75±0.02 |
| 46 | Pelargonidin 3-O-glucoside | 435.121 | 3.350 | 1.953 | 0.491 | -1.026 | 6.83±0.02 | 3.36±0.04 |
| 47 | 2,6-Pyridinedicarboxylic Acid | 200.057 | 2.862 | 2.034 | 0.489 | -1.031 | 5.86±0.06 | 2.87±0.02 |
| 48 | 8-Hydroxynevirapine glucuronide | 493.110 | 5.585 | 1.802 | 0.489 | -1.032 | 5.86±0.06 | 2.87±0.33 |
| 49 | Epicatechin 3-O-(3-O-methylgallate) | 439.105 | 5.047 | 2.015 | 0.489 | -1.032 | 6.16±0.10 | 3.02±0.90 |
| 50 | Histidylhistidine | 313.101 | 3.646 | 1.947 | 0.487 | -1.037 | 6.86±0.06 | 3.34±0.51 |
| 51 | Humilixanthin | 344.147 | 2.886 | 1.978 | 0.485 | -1.044 | 5.5±0.06 | 2.67±0.02 |
| 52 | Chrysin 7-[rhamnosyl-(1->4)-glucoside] | 583.140 | 3.991 | 1.856 | 0.483 | -1.049 | 6.34±0.17 | 3.07±0.73 |
| 53 | Lysyltyrosine | 292.167 | 5.079 | 1.922 | 0.482 | -1.053 | 5.55±0.05 | 2.67±0.89 |
| 54 | 3'-(2''-Galloylglucosyl)-phloroacetophenone | 500.143 | 2.988 | 2.138 | 0.482 | -1.054 | 6.38±0.08 | 3.07±0.02 |
| 55 | N-Phenethylacetamide | 164.108 | 5.430 | 2.147 | 0.477 | -1.069 | 6.37±0.06 | 3.04±0.09 |
| 56 | 2-Pentanone | 107.048 | 4.960 | 1.888 | 0.475 | -1.075 | 6.19±0.06 | 2.94±0.04 |
| 57 | Glucose lactate glutamate | 446.147 | 5.047 | 2.197 | 0.475 | -1.076 | 6.81±0.07 | 3.23±0.66 |
| 58 | 4-O-Methylgalactinol | 337.111 | 1.431 | 1.939 | 0.473 | -1.080 | 6.51±0.03 | 3.08±0.04 |
| 59 | N-Formyl-L-aspartate | 321.059 | 5.093 | 2.056 | 0.465 | -1.105 | 7.65±0.09 | 3.56±1.11 |
| 60 | Equol 7-O-glucuronide | 460.163 | 4.509 | 2.047 | 0.461 | -1.118 | 5.81±0.05 | 2.68±0.67 |
| 61 | 5'-Deoxyadenosine | 288.049 | 6.117 | 1.890 | 0.460 | -1.120 | 6.11±0.05 | 2.81±0.40 |
| 62 | Kentsin | 539.268 | 6.824 | 2.282 | 0.458 | -1.127 | 7.06±0.07 | 3.23±0.49 |
| 63 | 4-(Glutamylamino) butanoate | 271.071 | 1.266 | 2.265 | 0.456 | -1.134 | 6.99±0.03 | 3.18±0.64 |
| 64 | Artemisinin | 265.145 | 3.179 | 2.008 | 0.455 | -1.135 | 5.36±0.08 | 2.44±0.02 |
| 65 | Epsilon-Tocopherol | 393.317 | 7.507 | 2.388 | 0.449 | -1.157 | 7.49±0.1 | 3.36±0.09 |
| 66 | Trimethylamine N-oxide | 151.145 | 0.950 | 2.353 | 0.446 | -1.165 | 7.35±0.05 | 3.28±0.57 |
| 67 | Hydroxyaniline mustard | 214.019 | 1.994 | 1.980 | 0.444 | -1.171 | 6.43±0.05 | 2.86±0.04 |
| 68 | Oxynarcotine | 473.195 | 5.668 | 1.989 | 0.438 | -1.192 | 5.76±0.05 | 2.52±1.34 |
| 69 | Methyltrienolone | 586.385 | 6.442 | 2.204 | 0.436 | -1.198 | 6.23±0.08 | 2.72±0.02 |
| 70 | Azlocillin | 462.142 | 4.304 | 2.192 | 0.436 | -1.199 | 6.18±0.09 | 2.69±0.15 |
| 71 | Daidzin | 434.147 | 4.659 | 2.146 | 0.436 | -1.199 | 6.18±0.09 | 2.69±0.83 |
| 72 | Isopentenyladenine | 204.126 | 4.541 | 2.139 | 0.435 | -1.200 | 5.88±0.08 | 2.56±0.13 |
| 73 | Phenylalanylarginine | 304.179 | 3.099 | 2.080 | 0.433 | -1.209 | 5.56±0.07 | 2.41±0.30 |
| 74 | N-Acetyllactosamine | 420.090 | 5.530 | 1.997 | 0.431 | -1.215 | 6.39±0.05 | 2.75±0.10 |
| 75 | Djenkolic acid | 235.022 | 3.646 | 2.105 | 0.429 | -1.222 | 7.07±0.04 | 3.03±0.04 |
| 76 | Liquiritin | 460.163 | 4.611 | 2.199 | 0.424 | -1.237 | 6.14±0.05 | 2.6±0.38 |
| 77 | N6-(delta2-isopentenyl)-adenosine 5'-monophosphate | 460.121 | 4.811 | 2.087 | 0.418 | -1.260 | 6.85±0.09 | 2.86±0.24 |
| 78 | Mahuannin A | 581.088 | 5.921 | 2.067 | 0.408 | -1.292 | 6.59±0.07 | 2.69±0.08 |
| 79 | Pro-Pro-Pro | 310.178 | 2.830 | 2.113 | 0.408 | -1.293 | 5.64±0.10 | 2.30±0.70 |
| 80 | Silidianin | 465.114 | 5.780 | 1.937 | 0.401 | -1.317 | 5.92±0.05 | 2.38±0.71 |
| 81 | Cholestane-3,7,12,25-tetrol-3-glucuronide | 647.356 | 6.360 | 2.007 | 0.394 | -1.344 | 6.16±0.07 | 2.43±0.53 |
| 82 | N-[[3-(b-D-Glucopyranosyloxy)-2,3-dihydro-2-oxo-1H-indol-3-yl]acetyl]aspartic acid | 505.106 | 5.749 | 1.991 | 0.389 | -1.363 | 6.17±0.09 | 2.4±0.84 |
| 83 | Glycinoeclepin A | 447.240 | 6.243 | 2.214 | 0.389 | -1.364 | 5.89±0.08 | 2.29±0.47 |
| 84 | (-)-Wikstromol | 416.173 | 5.143 | 2.226 | 0.386 | -1.372 | 5.85±0.11 | 2.26±0.07 |
| 85 | (S)-a-Amino-2,5-dihydro-5-oxo-4-isoxazolepropanoic acid N2-glucoside | 315.080 | 3.319 | 1.941 | 0.382 | -1.388 | 5.64±0.11 | 2.16±0.48 |
| 86 | (22R,23R)-22,23-dihydroxy-campest-4-en-3-one | 395.333 | 6.935 | 2.194 | 0.380 | -1.396 | 5.62±0.11 | 2.14±0.04 |
| 87 | Deoxyloganin | 419.132 | 2.901 | 2.346 | 0.377 | -1.409 | 6.81±0.06 | 2.56±1.20 |
| 88 | Phenytoin methylcatechol | 279.078 | 6.116 | 2.180 | 0.376 | -1.411 | 5.53±0.10 | 2.08±0.24 |
| 89 | (6-Aminoquinolin-2-yl) N-(2,5-dihydroxypyrrol-1-yl)-N-hydroxycarbamate | 353.030 | 6.093 | 2.235 | 0.364 | -1.460 | 7.31±0.07 | 2.66±0.69 |
| 90 | Prodiamine | 385.092 | 6.187 | 2.281 | 0.360 | -1.476 | 7.79±0.06 | 2.80±1.23 |
| 91 | Dimethyl 2-oxoglutarate | 157.051 | 3.465 | 2.320 | 0.358 | -1.484 | 6.14±0.02 | 2.19±0.45 |
| 92 | 6-Methylnicotinamide | 318.193 | 3.179 | 2.207 | 0.357 | -1.488 | 5.66±0.07 | 2.02±0.76 |
| 93 | Cyclohexyladenosine | 388.141 | 4.968 | 2.275 | 0.349 | -1.520 | 6.61±0.07 | 2.30±1.95 |
| 94 | 2-Methylthio-N6-(delta2-isopentenyl)adenosine | 426.121 | 5.948 | 2.259 | 0.345 | -1.536 | 5.70±0.11 | 1.97±0.42 |
| 95 | (3beta,22E,24R)-Ergosta-4,6,8(14),22-tetraen-3-ol | 377.322 | 6.553 | 2.522 | 0.342 | -1.549 | 6.99±0.11 | 2.39±0.09 |
| 96 | Lacto-N-triaose | 609.211 | 5.111 | 2.322 | 0.331 | -1.597 | 5.83±0.07 | 1.93±0.12 |
| 97 | Fumarycarnitine | 258.096 | 5.741 | 2.204 | 0.311 | -1.683 | 6.46±0.09 | 2.01±0.31 |
| 98 | Trovafloxacin | 434.147 | 4.825 | 2.401 | 0.311 | -1.687 | 6.06±0.08 | 1.88±0.21 |
| 99 | Dihydrodaidzin | 460.163 | 4.857 | 2.375 | 0.310 | -1.692 | 5.94±0.05 | 1.84±0.36 |
| 100 | 2-Thiouric acid | 204.979 | 2.338 | 2.457 | 0.285 | -1.811 | 7.70±0.05 | 2.2±0.09 |
| 101 | Isohydroxymatairesinol | 392.173 | 5.517 | 2.265 | 0.267 | -1.908 | 5.67±0.07 | 1.51±1.62 |
| 102 | Mollicellin C | 430.153 | 5.956 | 2.353 | 0.255 | -1.974 | 5.62±0.06 | 1.43±1.04 |
| 103 | Amphibine H | 669.331 | 6.291 | 2.595 | 0.254 | -1.976 | 6.55±0.04 | 1.67±0.34 |
| 104 | (-)-Epicatechin 8-C-galactoside | 451.120 | 3.435 | 2.274 | 0.244 | -2.033 | 6.50±0.01 | 1.59±1.11 |
| 105 | Methyl (3x,4E,10R)-3,10-dihydroxy-4,11-dodecadiene-6,8-diynoate 10-glucoside | 361.130 | 5.119 | 2.391 | 0.232 | -2.105 | 5.39±0.08 | 1.25±0.02 |
| 106 | 17-beta-estradiol 3-sulfate-17-(beta-D-glucuronide) | 573.143 | 5.461 | 2.725 | 0.231 | -2.117 | 6.98±0.03 | 1.61±0.02 |
| 107 | Thiophen-2-ol | 259.011 | 0.942 | 2.189 | 0.225 | -2.152 | 5.67±0.06 | 1.28±0.34 |
| 108 | 3-Oxotridecanoylcarnitine | 392.240 | 6.453 | 2.165 | 0.225 | -2.153 | 5.52±0.09 | 1.24±0.03 |
| 109 | 2-Cyano-3-[3-ethoxy-4-hydroxy-5-[(phenylthio)methyl]phenyl]-2-propenamide | 399.101 | 3.748 | 2.440 | 0.216 | -2.212 | 7.16±0.05 | 1.55±1.19 |
| 110 | 5-Amino-2-oxopentanoic acid | 304.152 | 3.203 | 2.439 | 0.177 | -2.501 | 5.35±0.08 | 0.95±0.69 |
| 111 | Cyanidin 3-rutinoside | 616.135 | 3.490 | 2.261 | 0.161 | -2.632 | 5.56±0.05 | 0.90±0.03 |
| 112 | Isoleucyl-Lysine | 242.188 | 2.957 | 2.777 | 0.025 | -5.322 | 5.74±0.08 | 0.14±0.26 |
| 113 | 4-Hydroxy-5-(3',4',5'-trihydroxyphenyl)-valeric acid-O-methyl-O-glucuronide | 483.090 | 5.200 | 2.653 | 0.000 | -13.288 | 6.42±0.08 | 0.00±0.00 |

Note: MEAN in the table is the relative quantitative mean value of the substance in sample replicate experiments.
